# What drives slow wave activity during early non-REM sleep: Learning during prior wake or effort?

**DOI:** 10.1371/journal.pone.0185681

**Published:** 2017-10-13

**Authors:** Ziyang Li, Aarohi B. Sheth, Bhavin R. Sheth

**Affiliations:** 1 University of Houston, Houston, TX, United States of America; 2 Carnegie Vanguard High School, Houston, TX, United States of America; Waseda University, JAPAN

## Abstract

What is the function of sleep in humans? One claim is that sleep consolidates learning. Slow wave activity (SWA), i.e. slow oscillations of frequency < 4 Hz, has been observed in electroencephalograms (EEG) during sleep; it increases with prior wakefulness and decreases with sleep. Studies have claimed that increase in SWA in specific regions of the sleeping brain is correlated with overnight improved performance, i.e. overnight consolidation, on a demanding motor learning task. We wondered if SWA change during sleep is attributable to overnight consolidation or to metabolic demand. Participants executed out-and-back movements to a target using a pen-like cursor with their dominant hand while the target and cursor position were displayed on a screen. They trained on three different conditions on separate nights, differing in the amount and degree of rotation between the actual hand movement direction and displayed cursor movement direction. In the no-rotation (NR) condition, there was no rotation. In the single rotation (SR) condition, the amount of rotation remained the same throughout, and performance improved both across pre-sleep training and after sleep, i.e. overnight consolidation occurred; in the random rotation (RR) condition, the amount of rotation varied randomly from trial to trial, and no overnight consolidation occurred; SR and RR were cognitively demanding. The average EEG power density of SWA for the first 30 min. of non-rapid eye movement sleep after training was computed. Both SR and RR elicited increase in SWA in the parietal region; furthermore, the topographic distribution of SWA in each was remarkably similar. No correlation was found between the overnight performance improvement on SR and the SWA change in the parietal region on measures of learning. Our results argue that regulation of SWA in early sleep is associated with high levels of cognitive effort during prior wakefulness, and not just overnight consolidation.

## Introduction

Sleep has been demonstrated to play a critical role in overnight consolidation and memory consolidation for a diverse array of memories [1–13]. Sleep, in particular slow wave sleep (SWS), and the associated slow-wave activity (SWA) of less than 4 Hz [14], is known to be homeostatically regulated [15]. Thus, the activation of a brain region during performance and/or learning in the wake state could lead to the homeostatically driven induction of SWS and SWA during the subsequent sleep state at a local level in the brain. Studies have shown that sleep/ homeostasis does have a local component–in addition to a global one–which can be triggered by a learning task involving specific brain regions [16]. Experimental studies have claimed that overnight reactivation, or replay, of memories is a neural mechanism by which sleep drives the overnight consolidation of memories that involve the hippocampus in humans [5,17] and other species [18,19]. A second candidate neural mechanism is the synaptic homeostasis hypothesis [20]. According to this hypothesis, SWA in sleep is associated with synaptic downscaling or de-potentiation, which counteracts the effects of synaptic potentiation in wake, and enhances the consolidation of strong, useful memories by filtering out weaker, useless ones. The latter mechanism may be more relevant to memories that are not dependent on the hippocampus, e.g. motor memories.

On the other hand, homeostasis is a more general notion than one associated with memory alone. Sleep homeostasis could well be the result of general brain restitution arising from the demands of usage and resulting fatigue [21–23]. Notably, there is a large body of literature on induced local sleep that argues for sleep homeostasis not due to memory consolidation processes but, rather, to effort, engagement, and high levels of brain activity. Apropos, it has been found that adenosine and adenosine receptors, i.e. adenosinergic transmission, play a role in non-REM sleep homeostasis in humans [24]; adenosine concentration increases in the basal forebrain during wake, and this increase induces sleep [25]. The increase in adenosine concentration during sleep deprivation in localized cortical areas suggests that adenosine may have a role in the local regulation of sleep homeostasis. This could be achieved through astrocytes, which are known to modulate activity [26] (a process termed gliotransmission) in local neuronal assemblies, i.e. cortical columns, that enter a sleep-like state after a prolonged period of activity–activity corresponds to usage–of said assemblies [27]. In light of these studies, we can extend further the logic at the paragraph's beginning: Mechanisms purportedly linked to sleep homeostasis, i.e. SWA, especially early in non-REM sleep, are likely to be correlated not just with overnight consolidation but also, or rather, with the restitution of task-related brain circuits fatigued by taxing task demands and high levels of effort [7,8].

Thus, enhanced SWA in localized regions of the brain during early non-REM sleep could either be the result of memory consolidation, or be the result of effort during the prior wake period and recovery from its taxing metabolic and/or cognitive demands. Dissociating between these two possibilities is an important issue that is not yet fully resolved.

With these issues in mind, we decided to address the following questions in the present study. First, does the overnight consolidation of memory in cortical areas participating in performance on a given task trigger the putative local induction of SWA in these areas? If the answer to the question is affirmative, it would suggest that local plastic changes in the brain associated with memory consolidation may be involved, directly or indirectly, and will confirm past studies. On the other hand, if overnight memory consolidation is not found to be associated with local induction of SWA in the brain, then some other mechanism may be involved in locally inducing SWA in early sleep. Second, we asked if local SWA homeostasis is strongly correlated with improved performance following a night of sleep on a task that was learnt just before sleep. If the answer to the second question is affirmative, then it would suggest that SWA homeostasis is related to processes underlying memory consolidation; on the other hand, if the answer is negative, it would suggest that other processes, e.g. processes related to metabolic fatigue or recovery from cognitive and motor demand and usage etc., may be involved in addition or instead.

In order to address these questions, we designed a motor learning task that was similar to that used in past studies [16,28]. As in the aforementioned studies, participants reached for visual targets using a handheld cursor on a flat tablet while unconsciously adapting to systematic rotations imposed on the visually seen cursor trajectory. On different sessions conducted on the same set of participants 1–2 weeks apart, there was either no systematic rotation at all (no-rotation or NR) of the perceived cursor trajectory, a single systematic rotation (single rotation or SR) of the perceived cursor trajectory, or a trial-by-trial random variation in the direction (i.e. angle) of the perceived cursor trajectory (random rotation or RR). The results of the study provide tentative answers to the questions raised above. SWA increase was induced more strongly in certain parts of the brain than others—in particular, SWA increase was greater in the left parietal region, contralateral to the hand used to executed movements on the task; however, we did not find a significant degree of correlation between the amount of learning across a night of sleep and the local increase in SWA during this period. Instead, we found a similar localized increase in SWA in the contralateral parietal cortex on RR in which no learning occurred whatsoever; moreover, the overall pattern of SWA across the cortical surface in both the learning (SR) and no learning (RR) conditions was remarkably and significantly similar, suggesting mechanisms other than learning but nevertheless common to both, i.e. effort and cognitive demand, could be responsible for the underlying early pattern of SWA in the sleeping human brain.

## Materials and methods

### 1. Experiment design

#### 1.1. Motor task setup and apparatus

The motor tasks required participants to move a pen-like cursor with their dominant hand on a 12” X 18.2” digitizing tablet (Intuos4 XL, Wacom Inc.) while the target and the cursor position were displayed on a computer screen (gain: 1:1). Participants were required to sit facing the vertical computer screen with the tablet placed horizontally at waist height. An opaque shield was placed above the participant’s hand in order to prevent them from seeing their hand during the task. Two speakers were set up to play tones indicating the time period during which the participant had to execute and complete the motor action.

#### 1.2. Electroencephalography (EEG) and polysomnography (PSG)

Participants lay in a supine position in a comfortable bed in a noise reduced room (the room was electromagnetically shielded and attenuated ambient sound from the outside). Prior to that, a cap with EEG electrodes was placed over their heads for EEG recordings while they slept. EEG data were acquired on a 64+8 channel Active Two recording system (Biosemi, Inc.) at a sampling rate of 512 Hz. Note that high-density EEGs are not comfortable, and whole night recordings with the EEG cap were therefore not possible (see note A in S1 Appendix). Therefore, the cap was removed after approximately 2 hours of sleep and participants were then allowed to sleep undisturbed for the rest of the night, with no electrodes attached. All reported satisfactory sleep. EEG data acquired were pre-processed using a similar approach to that in previous studies [29]. The EEG data were filtered in MATLAB using Parks-McClellan FIR band-pass filters (0.5–50 Hz settings) and the MATLAB function filtfilt to minimize phase distortion. Independent components analysis (ICA) was then performed on the filtered EEG data. For this purpose, we made use of the EEGLAB toolbox [30] in MATLAB. We used the ICA decomposition algorithm in runica.m (Infomax) provided in EEGLAB. Artifacts such as eye movements, eye blinks, muscle artifacts, and irregular spikes, which are characterized by unique spectral and spatial signatures, were manually removed. It is important to note that the manual removal was performed with no regard paid to the underlying state of arousal (wake/sleep). Electromyography (EMG) data were filtered between 10 and 100 Hz. Electrooculography (EOG) data were filtered between 0.5 and 100 Hz. PSG scoring was given to a company (WideMed, Inc.); it utilized a software routine called Morpheus. The data were divided into 30-s epochs and each epoch was then scored automatically, based on standard PSG criteria [31]. The sleep stage scoring was manually verified by a human scorer.

#### 1.3. Characteristics of motor tasks

Participants ran three motor tasks on three separate nights at least one week apart. The tasks were similar in nearly all respects but differed in the ways each impacted learning and cognitive effort.

All motor tasks required out-and-back movements from a central starting area to one of eight targets (45° apart, distance of 6.5cm). Targets and starting area were 2cm diameter circles. Targets were colored solid blue and the starting area was colored white. Target locations were pseudo-randomized from trial to trial; on a single block of trials, the target was presented at each location an equal number of times. A trial block contained 50 successive trials (1 trial per second). For analysis purposes, the first and last trials were discarded and the remaining 48 trials were used.

A tone played 500 ms from trial onset, and lasted for 250 ms. Participants were asked to perform the out-and-back movements by following the tempo of the tones. A successful “hit” required the following: 1) the reversal point of each movement trajectory should be located within the target area (the reversal point is the farthest point from the center of the starting area); 2) reversal occurred within 375–625 ms from trial and target onset, i.e., the optimal performance time was to hit the target in the time the tone played; 3) the entire out-and-back movement was to be complete within 1-s of trial and target onset, i.e., the cursor had to be back in the starting area within 1-s. Only if all the three requirements were satisfied, would the trial be considered successful (and count as a hit), and the next trial be triggered on time. Note that the 1-s time requirement helps ensure the participant has to make a rapid movement with little time to cognate on the rotation angle. It bears mention in his regard that targets were highlighted at 1-s intervals in a previous study of visuomotor rotation adaptation [16].

We recorded movement trajectories as a function of time from trial and target onset. This allowed us to measure error (S1 Fig), using a measure similar to that used earlier [28]. The overall error measure combined errors from the out and back components of the movement and linear and directional errors. This combined measure allowed us to be sensitive to multiple different parts of a highly complex movement; therefore, improvement in any part of the movement ought to be reflected in the error measure.

#### 1.4. Experimental conditions

We devised three separate conditions. (1) Single-rotation (SR)–A single, fixed degree of rotation (+45°) was imposed between hand movement on tablet and cursor display on the screen. This condition was similar to that used in [16], which showed evidence for learning with training. (2) Random-rotation (RR)–From trial to trial, the degree of rotation imposed between hand movement and cursor display was varied pseudo-randomly (30°, 45°, 60°; the average rotation was 45^o^) across trials. There were 48 trials/block, and each rotation angle occurred on 8/48 trials of the block. We did not expect learning on this task. However, the condition was challenging and demanded participants’ attention and cognitive effort. (3) No-Rotation (NR) control–No rotation was imposed in this condition. Participants simply moved the cursor directly to the target and the cursor on the screen showed the true movement with no rotation. All participants, who have years of experience moving a computer mouse on a mousepad, performed this condition with high accuracy and minimal effort and attentional resources.

#### 1.5. Participants and experimental procedure

All studies were approved by the University of Houston Committee for the Protection of Human Subjects and all study participants provided written informed consent. Participants were required to have normal or corrected-to-normal vision and have no known learning disability. We determined eligibility by asking potential candidates whether they meet these criteria. Individuals with healthy sleeping habits and no self-reported history of mental disorders, drug use, or sleep disorders were included in our study. Individuals with abnormal sleep patterns (e.g. sleep apnea, periodic leg movements), as determined by an Epworth questionnaire, were excluded. Individuals with a self-reported history of epileptic seizure, autism, attention deficit disorder, illicit drug use, or dyslexia were excluded (they did not have to inform us of their specific history). Individuals with impaired vision were excluded as well. For this particular study involving overnight sleep in the laboratory, we had approval to recruit and run male participants.

Under these inclusion and exclusion criteria, fourteen volunteers by word of mouth to participate in the motor task. All were volunteers from the ECE department of UH, and were undergraduate students, visiting scholars, or graduate students who were classmates of ZL, and were recruited between June, 2010 and October, 2011. Of these, four dropped out because of they were unable to participate on all three motor tasks. Thus, in the end, ten right handed volunteers (all male, 23.3±4.2 years old; age range 18–31 years) ran on all three conditions, on three separate nights with a 1–2 week interval in between two successive conditions. Data and analysis from only these ten volunteers will be presented here. Participants used their dominant right hand to perform the tasks. The order of the three conditions was randomly varied across participants to control for order effects. No baseline demographic characteristics of the study population other than their age were recorded.

After running three blocks of practice trials on the NR control to familiarize themselves with the apparatus, the participant had to run 9 blocks of trials of the experimental condition (NR/SR/RR). Upon completion of the run, the participant slept in a noise-reduced room while we recorded their sleep (see **1.2** above for details). The recording was terminated after 2 hours and the participant was then allowed to continue to sleep for the rest of the night until their spontaneous awakening the next morning. Upon waking up, he ran another nine blocks on the given experimental condition.

### 2. Analysis

#### 2.1. Analysis of motor task performance

The trajectory of all the movements with time points was recorded, and for each block, the following parameters were computed: 1) overall error; 2) time duration; 3) onset time; 4) reversal time; 5) peak velocity.

Behavioral learning across sleep was measured as the normalized difference in the average errors between blocks 7–9 before sleep on the one hand and blocks 2–4 following sleep. Pre-sleep learning was measured as the normalized difference in the average errors between blocks 1–3 and blocks 7–9 (last three blocks) before sleep.

#### 2.2. Power spectral density (PSD) analysis and SWA

After bandpass filtering and clean-up of the EEG data as detailed in **1.2** above, power spectral density (PSD) of the first 30 minutes of non-REM sleep for each channel was calculated, in line with [16]. We used Welch's averaged, modified periodogram method (pwelch function in MATLAB), with the EEG data divided into 4-s long sections with 50% overlap and a Hann window (a Hann window affords low aliasing and a tradeoff of slightly lower frequency resolution) of the same length. We then computed the average PSD in the delta band (1–4 Hz), again in line with [16]; this corresponds to the strength of slow-wave activity (SWA) in the brain. The PSD at each recording channel thus computed was normalized over the average PSD of the reference in the delta band. The reference was the average PSD across all the EEG channels. Eq 1 summarizes the operations performed.

Pi,SWA=PSDf=1Hz,…,4Hz,i¯PSDf=1Hz,…,4Hz,i=1,…,n¯¯ 1)

where $\bar{\left(\right)}$ represents the averaging operator, *f* represents frequency, *i* represents EEG channel, *n* is the total number of recording channels/electrodes, *PSD*_*f* = 1*Hz*,…,4*Hz*,*i*_

Topographic distribution of percentage change in SWA between SR and NR conditions, i.e. (SR—NR) / NR, was computed, in accord with [16]; topographic distribution of percentage change in SWA between RR and NR conditions, i.e. (RR–NR) / NR, was computed as well.

#### 2.3 Correlation coefficient (*r*)

Next, we measured the similarity of the two normalized topographic distributions of SWA by computing the correlation coefficient (*r*) below using Eq 2 as shown below.

$$r=\frac{\sum_{i=1}^{n}\left({RR}_{i}-\bar{RR})({NR}_{i}-\bar{NR}\right)}{\sqrt{\left(\sum_{i=1}^{n}{\left({RR}_{i}-\bar{RR}\right)}^{2})(\sum_{i=1}^{n}{\left({NR}_{i}-\bar{NR}\right)}^{2}\right)}}$$ 2)

where *RR*_*i*_ and *NR*_*i*_ are normalized percentage change in SWA in EEG channel *i* on the *RR* and *NR* conditions respectively, $\bar{RR}$ and $\bar{NR}$ are the average normalized percentage change in SWA averaged across all EEG channels *i* = 1, …, *n*, on the *RR* and *NR* conditions respectively.

We used the MATLAB function corrcoef which outputs the correlation coefficient *r* and the p-value, which is the probability of getting a correlation as large as the observed value *r* by random chance. The p-value was computed by transforming the correlation to create a t-statistic having *n* − 2 degrees of freedom, where *n* is the number of EEG channels.

## Results

Polysomnography recordings after learning on each of the three conditions (NR, SR, and RR) showed the usual progression of sleep stages. Values (mean ± s.e.m.) for NR, SR, and RR were, respectively: sleep onset latency, 3.2 ± 1.6, 0.8 ± 0.6, and 0.9 ± 0.5 min; wakefulness 10.7 ± 2.2%, 11.3 ± 2.4% and 9.0 ± 1.8%, rapid eye movement (REM) sleep, 2.3 ± 1.6%, 2.6 ± 1.4% and 3.8 ± 2.1%; non-REM sleep stage 2, 48.2 ± 4.3%, 45.9 ± 5.7% and 51.7 ± 5.0%, non-REM slow-wave sleep or SWS (stages 3–4) 38.7 ± 5.4%, 40.2 ± 7.1% and 35.5 ± 5.8% (% of recording time). Note that the percentages are for the first 60 min. of recording only, and the low proportion of REM sleep and high proportion of SWS are to be expected.

### Motor behavioral data: learning across sleep in the single rotation (SR) condition alone

**Fig 1:**
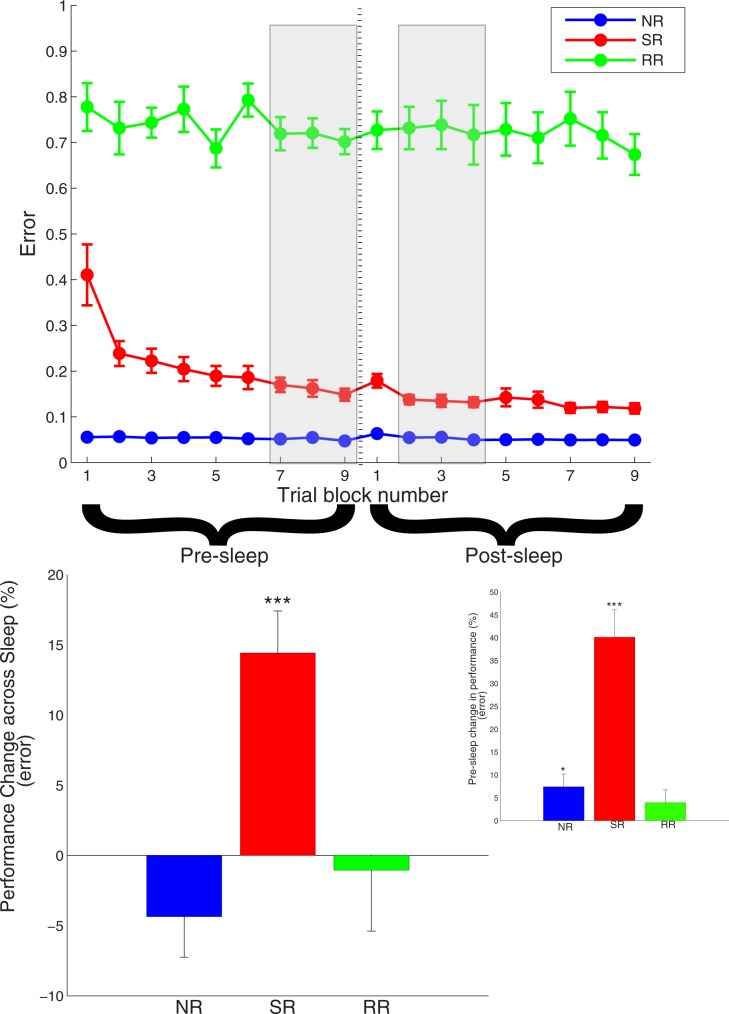
Motor performance across training and following sleep. (A) plots group mean error (ordinate) as a function of trial block number (1–9) before and after a night of sleep on the three conditions tested (SR: red; RR: green; NR: blue). Error bars are one standard error of the mean (s.e.m.). (B) plots percentage improvement in motor performance [(error_pre-sleep_−error_post-sleep_)/error_pre-sleep_] across a night of sleep. The error on pre-sleep trials 7–9 and post-sleep trials 2–4 (shown as gray shaded areas in Fig 1A) were averaged for each individual participant to obtain respectively pre-sleep and post-sleep values for each individual participant, following which the group mean and s.e.m. were calculated, which are displayed. (* = p < 0.05, ** = p < 0.01, *** = p < 0.005). Inset plots percentage improvement in motor performance across the pre-sleep session of training trials. Average errors on pre-sleep trial blocks 7–9 and pre-sleep blocks 1–3 were used to calculate percentage change.

Fig 1 (S2 Fig shows the same as Fig 1 but with colors friendly to color-blind readers) shows the error as a function of trial number for all three conditions–SR (red), RR (green), and NR (blue). Pre-sleep trials 1–9 are the training trials conducted on the first night of the given condition just before the participant went to sleep, and post-sleep trials 1–9 are test trials conducted the following morning after the participant had had a normal night of sleep (Fig 1A). In order to investigate learning across the night, we compared motor performance on trials 7–9 just prior to sleep and trials 2–4 immediately following sleep. Fig 1B shows that learning occurred, i.e. error decreased, only on the SR condition in which the cursor was rotated by a fixed angle of rotation across all (pre- and post-) trials of the condition. The overnight improvement in performance on the SR condition was significant (t(9) = 4.107, p = 0.003). Similar analyses on the RR and NR conditions did not find improvement in motor performance on either condition (RR: t(9) = -0.558, p = 0.590; NR: t(9) = -1.274, p = 0.235). Fig 1B (inset) shows change in motor performance, i.e. decrease in error, across training prior to sleep and shows that training led to significant decrease in motor error on the SR (t(9) = 4.711, p = 0.001) and NR (t(9) = 2.897, p = 0.018) conditions, but not on the RR condition (t(9) = 1.656, p = 0.132). In short, there was overnight improvement in motor performance on the SR condition alone, and reliable, significant improvement was not found on the RR condition, neither across training nor across a night of restful sleep (see S2–S5 Figs for graphs of other movement parameters as a function of block number before and following sleep).

### Local increase in SWA in sleep on single rotation condition and (lack of) correlation with overnight consolidation

**Fig 2:**
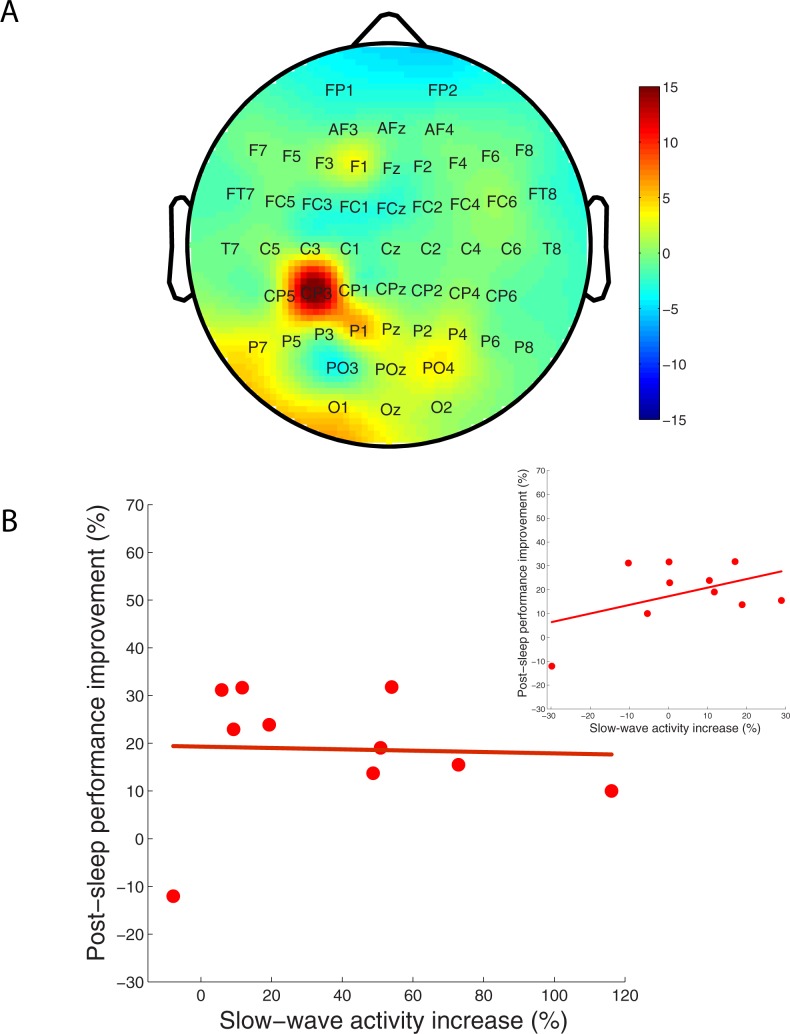
Topographic distribution of SWA during early non-REM sleep and correlation with learning. (A) shows the topographic distribution of the ratio of percentage change in slow-wave activity (SWA) during the first 30 min of non-REM sleep on the single rotation (SR) and the no-rotation (NR) conditions, i.e. SR/NR. Average EEG power density at 1–4 Hz (SWA, n = 10 participants) for the first 30 min of non-REM sleep was calculated on the SR and NR nights respectively in order to obtain the ratio, i.e. SR/NR, of change in power. Values obtained for each electrode were plotted at the corresponding position on the planar projection of the scalp surface, and interpolated between electrodes using the topoplot function provided in EEGLab. Warm colors indicate greater overnight increase in SWA during non-REM sleep on SR versus NR. (B) plots percentage improvement in motor performance [(error_pre-sleep_−error_post-sleep_)/error_pre-sleep_] across a night of sleep (ordinate) as a function of maximum increase in SWA (abscissa) across a nine electrode cluster located in the contralateral parietal cortex. Inset plots overnight percentage improvement in motor performance as a function of the average change in SWA across the same nine electrode cluster.

Fig 2A shows the topographic distribution averaged across all participants of the percentage change in SWA during the first 30 min of non-REM sleep between the single rotation (SR) and the no-rotation (NR) conditions. As the plot shows, large areas of the scalp showed an overall increase in SWA during the first 30 min of non-REM sleep following the learning. In particular–as indicated by the warm colors of the topoplot–the parietal brain region contralateral to the hand (all our participants were right-handed and used their dominant hand on the task) used on the motor task showed a large increase in SWA. We asked if the overnight increase in SWA in local regions of the brain coincided with overnight consolidation across the sample of participants. To address this question, we focused on a set of nine contiguous channels, i.e. CP5, CP3, CP1, P5, P3 P1, Pz, PO3, and POz, in the contralateral parietal region that showed the sharp SWA increase. There are bound to be individual variations in the exact location of the change in SWA across individual participants; we conducted a correlation analysis of increase in SWA vs. amount of overnight consolidation in two different ways to account for said inter-participant differences. In the first analysis, we correlated overnight consolidation with the site across the brain surface in each individual participant that showed the largest overnight SWA increase (Fig 2B). As the figure illustrates, there was no correlation between local increase in SWA during early non-REM sleep and overnight consolidation on the SR condition (r = -0.040, t(8) = 0.114, p = 0.912). In a companion analysis, we correlated overnight consolidation with the average (across the nine channels) change in SWA during early non-REM sleep (Fig 2B, inset). Again, the correlation between the two variables–learning and local increase in SWA–was not significant (r = 0.459, t(8) = 1.462, p = 0.182; also see note B in S1 Appendix). Combined, these analyses failed to demonstrate a significant correlation between overnight consolidation and local increase in SWA in the brain, casting doubt over the notion that local increase in SWA during early non-REM sleep has a causal effect on overnight consolidation.

### Topographic distribution of SWA on random rotation condition

**Fig 3:**
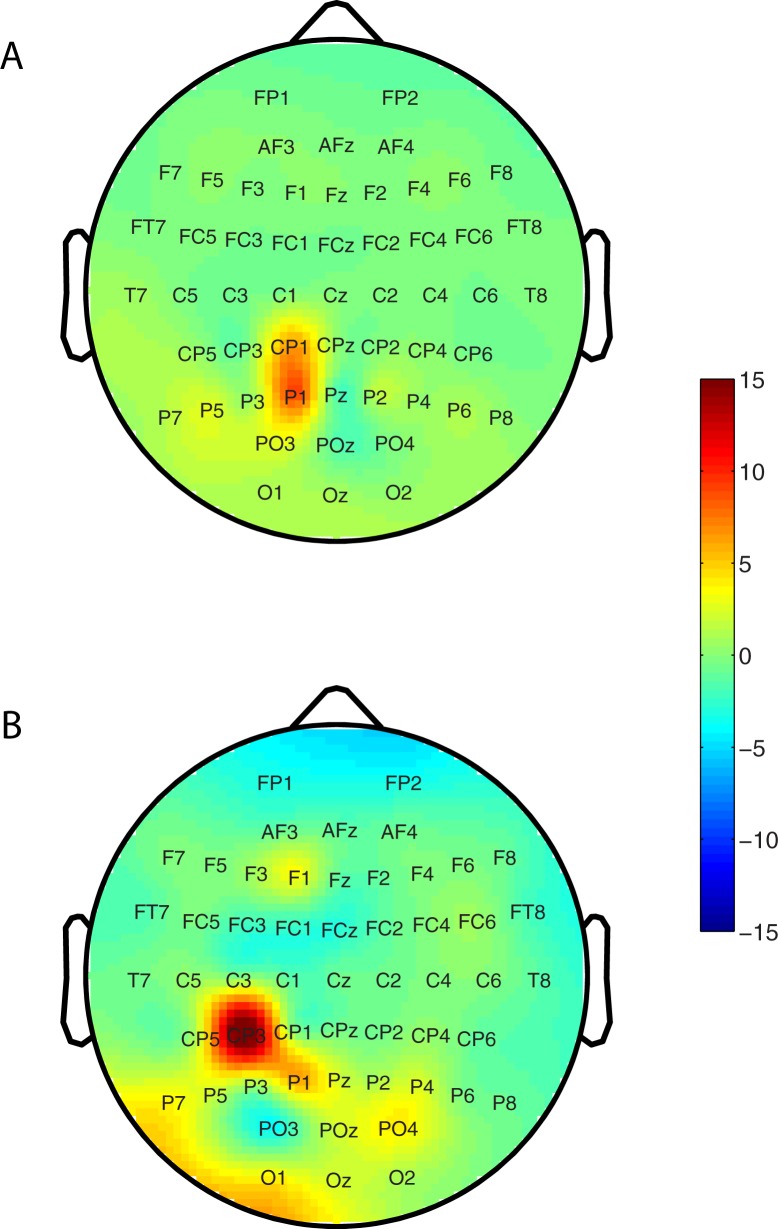
Group topographic distribution of SWA during early non-REM sleep on the random rotation (RR) and single rotation (SR) conditions. (A) shows the topographic distribution of the ratio of percentage change in SWA during the first 30 min of non-REM sleep on the random rotation (SR) and the no-rotation (NR) conditions, i.e. RR/NR averaged across the sample in our study. (B) is the same as Fig 2A and is shown here to facilitate the reader to make a direct comparison with Fig 3A.

**Fig 4:**
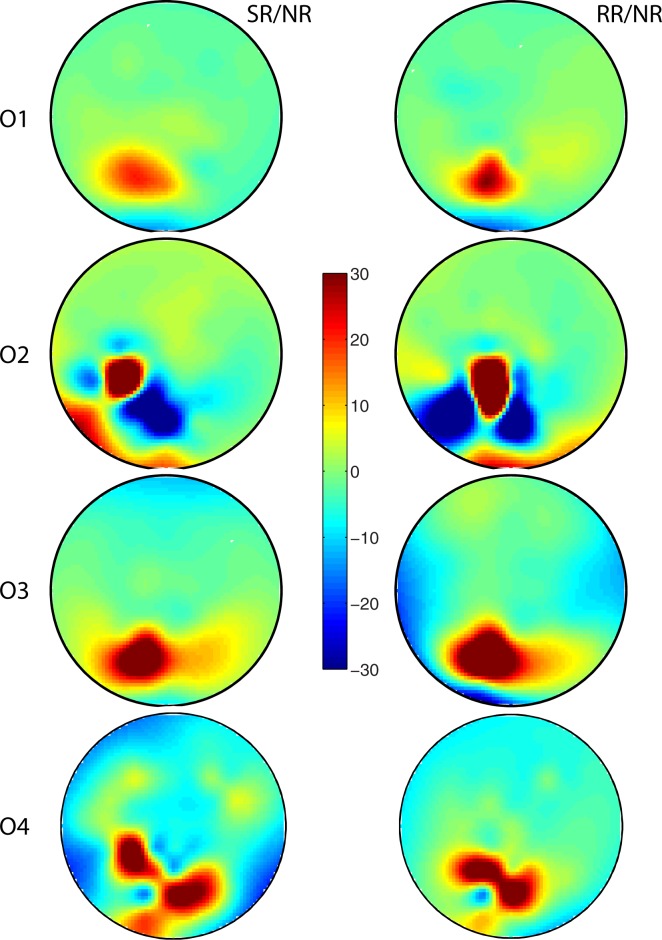
Topographic distribution of SWA during early non-REM sleep for individual participants. The figure shows the topographic distribution of the ratio of percentage change in SWA during the first 30 min of non-REM sleep on the normalized single rotation (SR/NR; left panel) and normalized random rotation (RR/NR; right panel) conditions for four representative participants in the study. Each row represents overnight SWA for an individual study participant.

We also measured SWA across the scalp in the RR condition on which no learning–overnight or over the course of training prior to sleep–was found. Fig 3A shows the mean topographic distribution of the percentage change in SWA during the first 30 min of non-REM sleep between the random rotation (RR) condition and the no-rotation (NR) baseline. There was an overall increase in SWA during this period with an especially large increase in the contralateral parietal cortex. Note that the analysis was identical to that used for comparing the topographic distribution of SWA change in SR/NR, which is illustrated again in Fig 3B (and Fig 2A) for the sake of a more direct comparison between the RR and SR conditions. At the group level, there was a small shift in the location of the largest SWA increase between the SR/NR and RR/NR cases, but overall, there was a remarkable degree of correlation across the surface, which was statistically significant (r = 0.463, p = 0.028). This was reflected at the level of individual participants: as Fig 4 shows, even though there was some variation across participants in the pattern of SWA during early non-REM sleep (Fig 4 shows topographic distributions from four representative participants), there was a remarkable degree of similarity in the topographic distributions across the scalp on the normalized SR (Fig 4, left panel) and RR (Fig 4, right panel) conditions (p < 0.05 for SR/NR vs. RR/NR correlation in 8/10 participants). Thus, across the surface, there were significant similarities in the topographic distribution of SWA in the normalized single rotation and random rotation conditions.

## Discussion

A previous, influential study [16] had participants perform a motor learning task in which they reached for visual targets using a handheld cursor while a systematic rotation was imposed on the perceived cursor trajectory. The study found a cluster of electrodes that showed increased SWA after rotation adaptation. The electrode cluster was located in the right, ipsilateral parietal lobe (in their study, right-handed participants used their dominant hand to perform the task), which differed from the location of the electrode cluster in the contralateral parietal region found in our study. We do not have a solid account for the discrepancy in the findings of the respective studies. However, we note that the previous study used a 256 channel system, more than the number of electrodes we used. Thus, it is possible that the apparently lower spatial resolution of our measurements may have failed to highlight the right, ipsilateral parietal region (On the other hand, given the effects of volume conduction, a 256 channel system does not necessarily have greater spatial resolution than a 64 channel one). However, we did have electrodes in said region and still did not find a substantial increase in SWA. On the other hand, we did find increased overnight SWA in the *contra*lateral parietal region, which is the brain area that is likely to be highly active when a participant is performing a motor learning task. A complementary EEG SWA study by the same group of researchers in which participants' left hand was immobilized during the day, also found a localized decrease in SWA during subsequent sleep in the *contra*lateral somatomotor cortex [32].

A second discrepancy is that in [16], the improvement in motor performance and increase in SWA in the local brain region (ipsilateral parietal region in their study) were positively correlated across their sample. On the other hand, we did not find a significant correlation between improvement in motor performance on SR and increase in local SWA (contralateral parietal region in our study) across our sample (maximum and average; see Fig 2B and 2B inset).

There is an important discrepancy between the adaptation protocols of the two studies that forms a basis for an alternative account of our findings, which we explore next. It assumes that participants used a cognitive strategy based on an explicit understanding of the amount of rotation. Here, we used a fixed rotation of 45° in the SR condition. The claim is that the participant was aware of the fixed 45° cursor rotation. When one is aware of such a rotation, adaptation to it is likely to occur with the selection of a cognitive strategy–in this case, the aware participant presumably pointed the cursor to the target -45^o^ from the actual target. The claim is that this learning is not a systematic, slow, implicit adaptation to a rotated display for the targets with the learning of a new visuo-motor map, but rather a fast process (lasting for a few movements and in any case no longer than a block) in which participants learn to apply a cognitive strategy or the same rule to all targets. Therefore, according to this viewpoint, our task is different from Huber et al's cursor rotation, which was implemented in smaller steps of 10 or 15° of which participants were not aware; this difference in task accounts for the difference in topography between the two studies. Further, the argument goes, the participant probably used similar strategies on the RR condition and explicitly learnt or guessed what to do, probably with more effort because of rotation unpredictability and increased use of visual feedback mechanisms for online corrections. Again, the account suggests that this supposed similarity in the SR and RR conditions accounts for the similarity in surface topography.

While the alternative possibility may appear attractive to some, there are several reasons that strongly argue against its feasibility. First, we did not inform the participants of a cognitive strategy to counter the visuomotor rotation. Thus, the participant would have had to figure out a cognitive strategy on their own while adapting to a brand new rotation that they had no idea about. This is unlikely to occur right away; if it were to occur, it would have to be after the participant acquires some skill on the task, forms a mental memory of the trajectory (s)he followed on the successful trials alone, and thus develops meta-cognition about the task and his/her performance–an altogether difficult proposition. Moreover, in a remarkable study on the relationship between implicit and explicit processes during visuomotor adaptation, investigators found that an implicit plan overrides an explicit strategy, even when instructions were provided to the subject to use an explicit, cognitive strategy [33]: While subjects in [33] were initially successful in completely negating the rotation with the instructed, explicit strategy, they were unable to sustain explicit control and made increasingly large errors to the desired target over the course of their task, to which they slowly and implicitly adapted later. The authors argued that the cognitive strategy failed because subjects simultaneously adapted unconsciously to the rotation to the neighboring target. Relevant to our study, they found that the rate of implicit adaptation to the target while a strategy was explicitly instructed was not significantly different from rotation adaptation when no explicit strategy was used or provided (see Fig 2 of [33])–the conditions in our task. Thus, even if explicit, cognitive strategies are present, they cannot substitute for implicit adaptation to a visuomotor rotation and are in fact overridden by the motor planning system. Even otherwise, not a single participant in our study expressed awareness of the rotation angle during or immediately after the task. We have since explicitly queried three participants and each denied using an explicit, cognitive strategy while training on the SR condition, and expressed surprise when informed that there was a fixed rotation angle across all targets on the SR condition. Finally, even if somehow the participant in our study somehow serendipitously stumbled upon a cognitive, explicit strategy–on their own and in spite of their denials that they used one–this form of learning is a fast process that lasts no longer than a block of trials (as the alternative account claims). However, as Fig 1 clearly shows and statistics confirm, adaptation continues beyond the first block as well as across the night, which is what was correlated with surface topography of SWA anyway. To quote [28]–was among the first studies to investigate visuomotor adaptation to a rotated display–"Since there was no explicit requirement for learning and since subjects were unaware of either change, this learning was ‘implicit’ in nature." In short, the alternative account of our findings is possible but unlikely (also see notes C and D in S1 Appendix for additional arguments against the alternative account). It is not entirely impossible, however, that the differences in adaptation protocols on the single rotation condition between our study and [16] accounts for the difference in topography, although there is–at least to our knowledge–no known reason why.

On the flip side of the argument above, our study replicated [16] in at least two key aspects: i) gradual improvement in motor performance, i.e. decrease in motor error, on the single rotation condition across both a session of learning and overnight across a period of restful sleep (see Fig 1); ii) SWA across the scalp during the early period of non-REM sleep showed an overall increase, and a particularly sharp one in a localized part of the parietal area (Fig 2). Thus, there are parallels between the two studies.

That we did not find a significant correlation between overnight consolidation and local SWA increase is not in line with learning or with the hypothesis that SWA during sleep is enhanced by replay and reactivation of memories acquired during the preceding wake period [5,18,34–36], but is with the idea that effort or usage during prior wakefulness drives SWA in subsequent sleep [15]. A more recent study conducted by the same group of researchers investigated the flip side of the issue: does reduced usage or effort reduce sleep need in the region corresponding to the effort [32]? The answer was yes: immobilizing a participant's arm during the day impaired motor performance of the immobilized arm, decreased evoked potentials over contralateral somatomotor cortex, and–of importance to us–reduced SWA in sleep over the same area. Thus, local sleep regulation occurred in the absence of learning in the classical sense, consistent with our findings. An EEG study on rats found that unilateral whisker stimulation during wake elicited a greater increase in SWA during non-REM sleep in the stimulated versus the control hemisphere [37]. The authors of the study concluded that regional SWA differences during sleep are use-dependent and could be related to the regional pattern of brain metabolism during prior wakefulness [37]. Because our results are in line with the aforementioned studies, it is not unreasonable to stipulate that the amount of overnight consolidation in our study is not perfectly correlated with greater expenditure of energy or cognitive effort (e.g. different individuals learn with differing degrees of effort and energy expenditure, which weakens whatever relationship exists between learning and energy/effort). Thus, local, homeostatic need does not necessarily have to be linked directly with learning, but could be more general. Our findings are consistent with the broader idea that SWA homeostasis reflects synaptic changes underpinning a cellular need for sleep [20], with the additional stipulation that said changes arise from cognitive demand and/or motor effort (cognitive demands occur when one performs a new, demanding cognitive/motor task, regardless of whether or how much one learns it). It must be emphasized, however, that our surface EEG studies, ipso facto, cannot directly address mechanistic questions at the cellular or synaptic level.

The results of our novel experimental manipulation, i.e. the random rotation condition, can be accounted for in this context. In particular, the topographical pattern of SWA across the scalp in study participants when they executed out-and-back movements under a non-systematic rotation of trajectory (RR condition) resembled significantly the topographical SWA pattern when they made movements under a systematic rotation of trajectory (SR condition), pooled over the population sample (Fig 3) as well as for a majority of individual participants (Fig 4). The remarkable similarity in SWA pattern between the two conditions signals a commonality between them: It is reasonable to conjecture that what underlies the similarity in brain pattern is that both tasks were similar in terms of attentional deployment, cognitive demand and/or motor effort, and both tasks actively engaged the brain (and fatigued the relevant brain circuits in so doing [7]). Studies have shown that a high cognitive load promotes homeostatic responses in sleep [38].

The synaptic homeostasis hypothesis [20] is relevant to our findings. The hypothesis is that wakefulness is associated with synaptic potentiation in cortical circuits, which, in turn, is tied to the homeostatic regulation of SWA; SWA is associated with synaptic downscaling or de-potentiation, which is tied to the beneficial effects of sleep on performance. Our EEG studies are at too high a level to be able to address cellular or synaptic aspects of the hypothesis, whether synaptic potentiation or de-potentiation occurs in wake, or even whether synaptic plasticity occurs on the random rotation condition. However, our findings are consistent with the idea that merely activity and effortful engagement with no concomitant learning is sufficient to modulate SWA in sleep. A recent animal study comes to a similar conclusion: Waking experience in a freely behaving rat–and neuronal activity corresponding to it–can cause neurons to briefly go "off-line", which is characterized by slow waves in the EEG [39], suggesting that intense waking activity can lead to SWA even when there is no learning.

It bears mention that our results showing a remarkable and significant similarity in the topographical distributions of SWA over the cortical surface between the SR and no-learning RR conditions do not argue that there is no contribution whatsoever for overnight consolidation and memory reactivation in SWA early in sleep. After all, the correlation between topographical distributions on the SR and RR conditions, while significant, was not one, indicating that there may be some pattern of distribution of SWA that is specific to learning; this will require careful probing to uncover in a future study that overcomes some of the limitations of the present one including, but not limited to, our small sample size, the lack of female participants, SWA recordings for only the early part of sleep and not the entire night, the absence of whole night PSG measurements, and a lack of an independent quantitative assessment of effort and/or attention.

## Conclusions

We investigated the effect of learning versus effort/engagement during wake on subsequent early non-REM sleep on a cognitively demanding motor task. We found no significant correlation between learning and local increase in early-night SWA in the surface EEG, and a topographical pattern in the surface distribution of early-night SWA that is associated with effort and cognitive demand during prior wake. Our results support a role for (early non-REM) sleep homeostasis in recovery from effort/engagement and not learning.

## Supporting information

S1 Appendix Notes. The notes provide supplementary information and arguments to the interested reader.
(DOCX)

S1 Fig Overall error. The overall error averages three distances: a, b and c shown in the figure. a is the distance between target and reversal point, b is the shortest distance between target and the outward movement trajectory, c is the shortest distance between target and inward (return) movement trajectory.
(EPS)

S2 Fig Motor performance across training and following sleep. (A) plots group mean error (ordinate) as a function of trial block number (1–9) before and after a night of sleep on the three conditions tested (SR: grey circles; RR: white circles with black frame; NR: black circles). Error bars are one standard error of the mean (s.e.m.). (B) plots percentage improvement in motor performance [(error_pre-sleep_−error_post-sleep_)/error_pre-sleep_] across a night of sleep (SR: grey bar; RR: white bar; NR: black bar). The error on pre-sleep trials 7–9 and post-sleep trials 2–4 (shown as light gray shaded areas in S2A Fig) were averaged for each individual participant to obtain respectively pre-sleep and post-sleep values for each individual participant, following which the group mean and s.e.m. were calculated, which are displayed. (* = p < 0.05, ** = p < 0.01, *** = p < 0.005). Inset plots percentage improvement in motor performance across the pre-sleep session of training trials. Average errors on pre-sleep trial blocks 7–9 and pre-sleep blocks 1–3 were used to calculate percentage change.
(EPS)

S3 Fig Total time duration. The graph plots group time duration (ordinate) as a function of trial block number (1–9) before and after a night of sleep on the three conditions tested (SR: red; RR: green; NR: blue). Error bars are one standard error of the mean (s.e.m.). Inset plots change across a night of sleep.
(EPS)

S4 Fig Onset time. The graph plots onset time (ordinate) as a function of trial block number (1–9) before and after a night of sleep on the three conditions tested (SR: red; RR: green; NR: blue). Error bars are one standard error of the mean (s.e.m.). Inset plots change across a night of sleep.
(EPS)

S5 Fig Reversal time. The graph plots reversal time (ordinate) as a function of trial block number (1–9) before and after a night of sleep on the three conditions tested (SR: red; RR: green; NR: blue). Error bars are one standard error of the mean (s.e.m.). Inset plots change across a night of sleep.
(EPS)

S6 Fig Peak velocity. The graph plots peak velocity (ordinate) as a function of trial block number (1–9) before and after a night of sleep on the three conditions tested (SR: red; RR: green; NR: blue). Error bars are one standard error of the mean (s.e.m.). Inset plots change across a night of sleep.
(EPS)
