# Supplementary material for: What drives slow wave activity during early non-REM sleep: Learning during prior wake or effort?
Source: PLoS One. 2017 Oct 13;12(10):e0185681. doi: 10.1371/journal.pone.0185681 (PMC5640223; doi:10.1371/journal.pone.0185681)
Supplement: S1 Appendix — The notes provide supplementary information and arguments to the interested reader. (DOCX) [file pone.0185681.s001.docx]

**S1 Appendix**

We intended to record all 64 EEG channels for each participant, but due to electrodes abruptly malfunctioning and subject movements during sleep, we could not record all 64 channels and a variable number of channels was recorded for each individual participant (55 channels on average).

Note that the outlier on the left that did not improve overnight and showed a strong SWA decrease during early non-REM sleep was largely responsible for shifting the correlation to a numerically (but not statistically) positive value.

There are other arguments against the alternative account. The alternative account, which is designed to explain why our study found similar surface topography on the SR and RR conditions, is based on the claim that the participant used similar cognitive strategies on both. We do not see a reasonable basis for this claim. If a similar cognitive strategy were used on RR, some learning, i.e. decrease in error, should occur on RR (albeit smaller and slower than that on SR). However, as Fig 1 shows, there was no decrease in error on RR at all. Apropos, our overall error measure (S1 Fig), because it combines three separate changes in the trajectory, is sensitive to small improvements; yet there was no discernible improvement on RR.

Fig 1A shows that error decreased by ~50% in the first block, which leads the proponents of the alternative account to argue that ours is not a systematic, slow, implicit adaptation, but rather a fast, explicit one. However, this initial rapid decrease in error is not unusual – cf. the learning curve illustrated in Fig 1b of [16], which showed an error decrease of >50% on the first block of each of 15^o^, 30^o^, 45^o^, and 60^o^ rotation directions. Moreover, [28] clearly states that adaptation to a rotated display is a type of implicit learning which occurs in two main phases: in the first phase, error falls rapidly, while the second phase is characterized by slower decrement in mean error. Indeed, our results dovetail nicely with [16] and [28]: as our Fig 1 shows, error continued to decrease in a more gradual fashion following the first block – prior to sleep as well as overnight.
